# Impact of Acne Vulgaris and Vitiligo on Quality of Life and Self-Esteem in the Patient Population of Madinah, Saudi Arabia

**DOI:** 10.7759/cureus.52586

**Published:** 2024-01-19

**Authors:** Samah O Alfahl, Lamiaa A Almehmadi, Ranad S Alamri, Dalia S Almalki, Fatimah Alnakhli

**Affiliations:** 1 Family Medicine, College of Medicine, Taibah University, Madinah, SAU; 2 Medicine, College of Medicine, Taibah University, Madinah, SAU; 3 Medicine, College of Medicine, Taibah University, Jeddah, SAU

**Keywords:** vitiligo, self-esteem, saudi arabia, dermatology life quality index, acne vulgaris

## Abstract

Background: Acne vulgaris and vitiligo are skin disorders that can have a negative impact on a person's self-esteem and quality of life (QoL). The purpose of this study is to look into the impact of acne and vitiligo on the self-esteem and QoL of the patient population in Madinah, Saudi Arabia.

Methods: A cross-sectional survey of 171 Saudi adults (141 individuals with acne vulgaris (9.4%) and 30 with vitiligo (1.5%) between the ages of 16 and 35 was conducted in Madinah. A self-reported questionnaire with four domains was used: socio-demographic data, lifestyle and coexisting pathologic factors or diseases, Rosenberg's self-esteem scale, and the dermatology life quality index (DLQI).

Results: Acne patients had a mean total score of 20.3 on the self-esteem scale, with 5% (n = 7) having low self-esteem, 48.2% (n = 68) having medium self-esteem, and 46.8% (n = 66) having high self-esteem. Females had higher self-esteem (75.29) than males (56.95). The mean DLQI score for acne patients was 5.4, with 30.5% (n = 47) unaffected, 29.1% (n = 45) mildly affected, 23.4% (n = 35) moderately affected, 5.6% (n = 10) severely affected, and 1.4% (n = 4) severely affected. Vitiligo patients had a mean self-esteem scale score of 13.7, with 63.3% (n = 19) having low self-esteem, 30% (n = 9) having medium self-esteem, and 6.7% (n = 2) having high self-esteem. The mean DLQI was 15.2, with 6.7% (n = 2) reporting that vitiligo had no effect on their lives, 10% (n = 3) reporting a moderate effect, 66.7% (n = 20) reporting a severe effect, and 16.7% (n = 5) reporting a very severe effect.

Conclusions: Our research confirms that acne vulgaris and vitiligo have a negative impact on self-esteem and quality of life. Along with medical treatment, effective treatment and psychological improvement of the patient should be prioritized.

## Introduction

The skin is an important organ for communicating with the outside world in order to perceive feelings, sexuality, and social interactions [1]. As a result, dermatological diseases, particularly visible ones that affect one's external appearance, are likely to have an impact on a patient's quality of life (QoL) and self-esteem. Acne vulgaris and vitiligo are two examples of psychodermatologic disorders that, like psoriasis or eczema, have a significant psychosocial impact on many people's lives. Skin conditions such as vitiligo and acne vulgaris frequently appear in childhood or adolescence and have a negative impact on patient's perceptions of their own bodies, which are inextricably linked to how they view themselves and interact with others. Furthermore, it may have a negative impact on their QoL [2].

Acne vulgaris is a chronic skin condition that affects the pilosebaceous unit. It is characterized by papules, pustules, nodules, cysts, and comedones and can be inflammatory or non-inflammatory. Acne vulgaris affects approximately 9.4% of the global population, with adults being the most affected [3,4]. The most common locations are the face, upper chest, and upper back [4]. The pathogenesis of acne vulgaris includes seborrhea, follicular inflammation, hyperkeratinization, and *Propionibacterium* acne colonization [5]. Acne vulgaris typically appears during puberty and can last until the age of 25. Even if acne vulgaris does not physically impair a person, it frequently causes post-inflammatory hyperpigmentation and scarring, which can carry a heavy psychological burden such as increased anxiety, anger, depression, and frustration. These emotions can then affect academic and professional performance, QOL, and self-esteem [3,4,6].

Vitiligo is a multifactorial, progressive, pigmented skin disease characterized by depigmented patches and macules caused by melanocyte loss. Vitiligo affects between 0.5% and 2% of the world's population [7]. The most common locations for vitiligo lesions on the body are the face, trunk, groin, axillae, and genitalia. Lesions can, however, appear on other parts of the body, such as the ankles, elbows, and knees. Vitiligo is most common in people between the ages of 10 and 30. It is one of the most psychologically devastating dermatological disorders, affecting not only psychological health but also several psycho-social aspects such as depression, anxiety, stigmatization, loss of self-beauty, physical deformity, loss of self-esteem, social discrimination, and all domains of QoL [8,9].

Self-esteem is defined as a person's perception of themselves. Positive self-esteem is a state in which an individual is aware of his or her positive characteristics, such as sufficiency, effectiveness, diligence, value, and success. Beauty, aesthetics, and pigmentation are highly valued in many cultures and societies worldwide. Any condition that affects one's appearance results in a loss of privileges, resources, and societal upward mobility. Because skin conditions are so visible, they frequently harm self-esteem [10].

Quality-of-life assessments are an important tool for identifying psychosocial issues in patients with chronic diseases. This is significant in terms of care commitment and chronic illness. The dermatology life quality index (DLQI) is a personal satisfaction measurement that is used to evaluate QoL, particularly for skin problems. Many studies [10] use this method to assess the health status of people with dermatological illnesses, including specialized and general skin disorders.

Previous global studies of QoL and self-esteem in patients with various skin diseases such as psoriasis, eczema, acne, vitiligo, and cutaneous melanoma revealed varying degrees of QoL impairment and self-esteem. In Saudi Arabia, patients with skin diseases have varying levels of QOL impairment and self-esteem. Many methodological issues, including the issue of cultural differences when the same measure is used in different countries, remain [6]. No previous community-based studies have been conducted on the impact of skin disease on QoL and self-esteem in Madinah, Saudi Arabia. This study aims to look at the self-esteem and QoL of acne vulgaris and vitiligo in the patient population of Madinah.

## Materials and methods

Study design and inclusion/exclusion criteria

This cross-sectional questionnaire-based study was conducted at Madinah between October 2021 and October 2022. The manual survey was conducted on the Saudi population over three months. The sample consisted of Saudi participants aged 16 to 35 of both sexes, diagnosed with acne vulgaris or vitiligo, and living in Medinah. Patients with any medical conditions (including HTN, diabetes mellitus (DM), asthma, cancer, epilepsy, or kidney failure) or other dermatological illnesses (including eczema, psoriasis, dermatitis, or alopecia) were excluded from the study. Patients with a history of psychiatric disorders (such as depression, bipolar disorder, anxiety disorder, panic attacks, psychotic disorder or schizophrenia, post-traumatic stress disorder, or attention deficit hyperactivity disorder) were also barred from participating.

Sample size calculation

In this study, two samples were present, i.e., one for acne and the other for vitiligo. Samples were calculated on the OpenEpi website. The first sample was for acne. We assumed the following: anticipated % frequency (p) 9.4, confidence level 95%, confidence limits +/- 5. The second sample was for Vitiligo. We assumed the following: anticipated % frequency (p) 1.5, confidence level 95%, confidence limits +/- 5.

Measurements

For the data collection, we used a self-reported web questionnaire in Arabic consisting of 36 close-ended questions. The questionnaire includes four important domains: socio-demographic data, lifestyle and coexisting pathologic factors or diseases, self-esteem, and QoL. It took 10 minutes for the participants to complete the three sections of the questionnaire.

The first section assesses the sociodemographic factors of patients (age, sex, nationality, social status, educational level, occupation status, income, and area of residence). The second section assesses self-esteem using the Arabic version of Rosenberg’s self-esteem scale (RSES), which is a 10-item scale that measures global self-worth by measuring both positive and negative feelings about the self. All items were answered using a 3-point Likert scale format, ranging from strongly agree to strongly disagree. Scores were calculated as follows for items 1, 3, 4, 7, and 10: strongly disagree = 0 points, disagree = 1 point, agree = 2 points, and strongly agree = 3 points. For items 2, 5, 6, 8, and 9 (which were reversed in valence), strongly disagree = 3 points, disagree = 2 points, agree = 1 point, and strongly agree = 0 points. The total score ranges from 0 to 30. The scoring system for the self-esteem scale is as follows: 0 to 14 = low self-esteem; 15 to 20 = moderate self-esteem; and 21 to 30 = high self-esteem.

The third section assesses QoL using the self-administered, Arabic version of the DLQI, a dermatology-specific questionnaire that assesses and evaluates health-related quality of life (HRQoL) in patients with dermatological diseases, especially adults. The DLQI consists of 10 items arranged in six categories: symptoms and feelings (questions 1 and 2), daily activity (3 and 4), leisure (5 and 6), work or study (7), interpersonal relationships (8 and 9), and treatment (10). The questions evaluate an individual’s perception of the disease over the past week. The 10 items of the questionnaire were rated on a 4-point scale: not at all or nothing relevant = 0, a little = 1, a lot = 2, and very much = 3. The DLQI was calculated by summing the scores of each question, resulting in a total score of 0 to 30. A higher total score represents a greater impairment of HRQoL. The DLQI scores are interpreted from the sum of the indices of the 10 items evaluated. The scoring system was categorized into five levels: no impairment = 0 to 1, mild impairment = 2 to 5, moderate impairment = 6 to 10, severe impairment = 11 to 20, and very severe impairment = 21 to 30.

Variables and validity

The independent variables were the occurrence of acne or vitiligo, age, and gender. The dependent variables were the impact of the acne or vitiligo on the patient's self-esteem and quality of life. An international score to assess the impact of QoL and self-esteem in patients with acne or vitiligo was used. It included items that clearly and adequately covered the domain of the content addressed.

Reliability analysis and pilot study

Reliability analysis was conducted on the total score on RSES and the DLQI. The values for Cronbach’s alpha were 0.83 for RSES and 0.88 for DLQI. All coefficients showed high and accepted reliability. After the development of the semi-structured questionnaire, a pilot study was carried out on 10 people to explore if any ambiguities or items led to misunderstanding the questionnaire. The feedback from the pilot study was incorporated for the questionnaire to reach its current final form.

Statistical analysis

The statistical software SPSS Statistics version 26.0 (IBM Corp., Armonk, NY, USA) was used for data analysis. Categorical variables were represented as frequency and percentages. Data normality was tested using the Kolmogorov-Smirnov test. Comparisons of participants' mean total score of the RSES and QoL by DLQI based on demographic variables were conducted using the T-test, Mann-Whitney U test for two groups, and the one-way ANOVA and Kruskal-Wallis H test for more than two groups. Cronbach’s alpha coefficients were computed to evaluate the internal consistency of RSES and the DLQI. A p-value of less than 0.05 was considered statistically significant, and the confidence interval was 95%.

## Results

Results of the acne vulgaris sample

This study included 141 Saudi acne vulgaris patients. Table 1 shows the demographic characteristics of the patients: females outnumbered males (76.6% (n = 108) versus 23.4% (n = 33)). The majority of participants (43.3%, n = 61) were between the ages of 21 and 25, single (75.2%, n = 106), students (66.7%, n = 94), and had an annual income of less than 3500 Saudi riyals (SR) (70.9%, n = 100). Approximately 36% (n = 51) of the patients live west of Madinah. Furthermore, 94.3% (n = 133) of patients were nonsmokers, and more than 62% (n = 88) visited a medical clinic.

**Table 1:** Sociodemographic characteristics of the acne vulgaris sample (n = 141) SR: Saudi riyal

Characteristics	Frequency	Percentages
Gender		
Male	33	23.4%
Female	108	76.6%
Age group		
16 to 20 years	50	35.5%
21 to 25 years	61	43.3%
26 to 30 years	30	21.3%
Social status		
Single	106	75.2%
Married	35	24.8%
Educational level		
Secondary education	45	31.9%
University	96	68.1%
Occupation status		
Student	94	66.7%
Employee	21	14.9%
Unemployed	26	18.4%
Income		
Less than 3500 SR	100	70.9%
3500-6000 SR	16	11.3%
6500-8000 SR	12	8.5%
More than 8000 SR	13	9.2%
Area of residence		
North of Madinah	13	9.2%
South of Madinah	16	11.3%
East of Madinah	28	19.9%
West of Madinah	51	36.2%
Central Region	33	23.4%
Clinic visit		
No	53	37.6%
Yes	88	62.4%
Smoking		
No	133	94.3%
Yes	8	5.7%

Table 2 describes the number and percentage of acne patients according to their responses on RSES. The mean of the total score for the sample on the scale was 20.3 (SD±4.1).

**Table 2:** Responses from and the mean for the acne vulgaris sample per the RSES (n = 141) RSES: Rosenberg’s self-esteem scale

Statements	Strongly agree		Agree		Disagree		Strongly disagree	
	N	%	N	%	N	%	N	%
1. On the whole, I am satisfied with myself.	38	27%	92	65.2%	11	7.8%	0	0%
2. At times I think I am no good at all.	5	3.5%	54	38.3%	54	38.3%	28	19.9%
3. I feel that I have a number of good qualities.	69	48.9%	67	47.5%	3	2.1%	2	1.4%
4. I am able to do things as well as most other people.	63	44.7%	69	48.9%	9	6.4%	0	0%
5. I feel I do not have much to be proud of.	3	2.1%	23	16.3%	67	47.5%	48	34%
6. I certainly feel useless at times.	10	7.1%	66	46.8%	0	0%	65	46.1%
7. I feel that I'm a person of worth, at least on an equal plane with others.	51	36.2%	74	52.5%	11	7.8%	5	3.5%
8. I wish I could have more respect for myself.	58	41.1%	65	46.1%	8	5.7%	10	7.1%
9. All in all, I am inclined to feel that I am a failure.	0	0%	13	9.2%	78	55.3%	50	35.5%
10. I take a positive attitude toward myself.	49	34.8%	79	56%	12	8.5%	1	0.7%
The mean of the total score (±SD)	20.3 (±4.1)							

**Figure 1:**
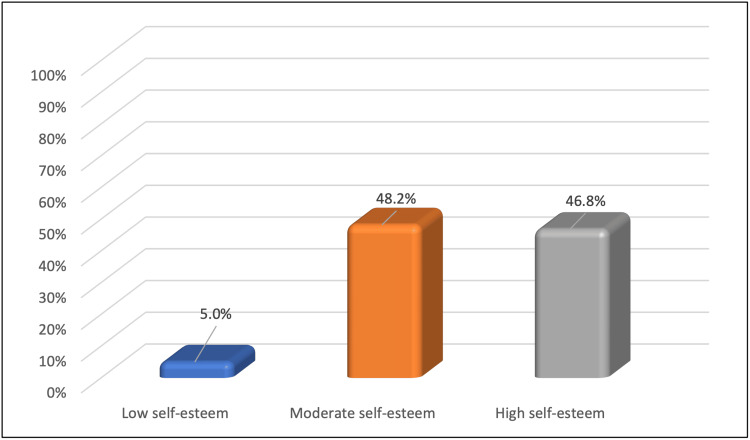
Levels of self-esteem in the acne vulgaris sample (n = 141)

Figure 1 shows the distribution of the levels of self-esteem in the acne vulgaris sample. Around 5% (n = 7) of the participants had low self-esteem, while 48.2% (n = 68) of them had a medium rating and 46.8% (n = 66) had high self-esteem.

Table 3 shows the responses of acne vulgaris patients per the DLQI. The mean of the total score for the sample on the scale was 5.4 (SD±5.1).

**Table 3:** Responses and the mean of the acne vulgaris sample per the DLQI (n = 141) DLQI: Dermatology life quality index

Questions	Very much		A lot		A little		Not at all OR Not relevant	
	N	%	N	%	N	%	N	%
Over the last week, how itchy, sore, painful or stinging has your skin been?	2	1.4%	12	8.5%	53	37.6%	74	52.5%
Over the last week, how embarrassed or self-conscious have you been because of your skin?	10	7.1%	31	22%	54	38.3%	46	32.6%
Over the last week, how much has your skin interfered with you going shopping or looking after your home or garden?	8	5.7%	8	5.7%	37	26.2%	88	62.4%
Over the last week, how much has your skin influenced the clothes you wear?	13	9.2%	26	18.4%	28	19.9%	74	52.5%
Over the last week, how much has your skin affected any social or leisure activities?	9	6.4%	27	19.1%	31	22%	74	52.5%
Over the last week, how much has your skin made it difficult for you to do any sport?	2	1.4%	6	4.3%	20	14.2%	113	80.1%
Over the last week, how much has your skin created problems with your partner or any of your close friends or relatives?	4	2.8%	15	10.6%	27	19.1%	95	67.4%
Over the last week, how much has your skin caused any sexual difficulties?	1	0.7%	0	0%	1	0.7%	139	98.6%
Over the last week, how much of a problem has the treatment for your skin been, for example by making your home messy, or by taking up time?	5	3.5%	23	16.3%	29	20.6%	84	59.6%
Over the last week, has your skin prevented you from working or studying?	No/Not relevant				Yes			
	140		99.3%		1		0.7%	
If "yes", over the last week how much has your skin been a problem at work or studying?			2	1.4%	17	12.2%	121	86.4%
The mean of the total score (±SD)	5.4 (± 5.1)							

**Figure 2:**
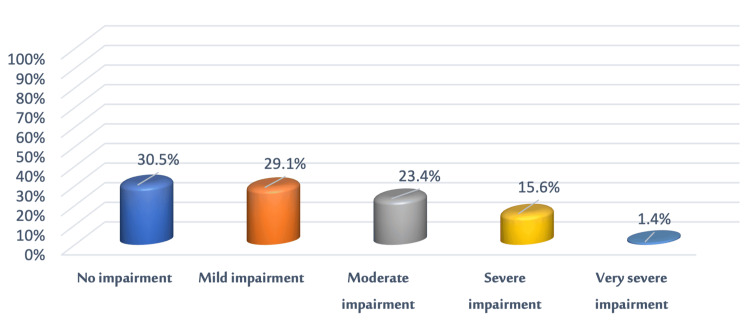
Levels of QoL in the acne vulgaris sample (n = 141) QoL: Quality of life

Figure 2 shows the distribution of the acne vulgaris sample to QoL levels per the DLQI. Of the patients, 30.5% (n = 43) reported that acne did not affect their lives, while 29.1% (n = 41) admitted that it had a mild effect on their lives, 23.4% (n = 32) said it had a moderate effect, 5.6% (n = 7) revealed it had a severe effect on their lives, and only 1.4% (n = 1) of patients reported that acne had a very severe effect on their lives.

Inferential statistics, i.e., the Mann-Whitney U and Kruskal-Wallis H tests, were applied to compare scores on the RSES with various demographic variables of the acne vulgaris sample. The results of the scale are shown in Table 4. There was a significant difference between the mean scores of males and females. Females showed higher self-esteem (75.29) than males (56.95).

**Table 4:** The differences between mean scores of RSES among demographic variables (n = 141) ^a^ Mann-Whitney U test, ^b^ Kruskal-Wallis test, p-value < 0.05 is statistically significant RSES: Rosenberg’s self-esteem scale, SR: Saudi riyal

Variables	Category	Mean rank	p-value
Gender ^a^	Male	56.95	0.024
	Female	75.29	
Age ^b^	16 to 20	70.87	0.990
	21 to 25	70.66	
	26 to 30	71.92	
Social status^ a^	Single	70.40	0.759
	Married	72.83	
Educational level ^a^	Secondary education	66.21	0.339
	University	73.24	
Occupation status ^b^	Student	74.39	0.314
	Employee	68.21	
	Unemployed	61.00	
Income ^b^	Less than 3500 SR	72.52	0.075
	3500-6000 SR	75.44	
	6500-8000 SR	42.42	
	More than 8000 SR	80.23	
Area of residence^ b^	North of Madinah	69.31	0.991
	South of Madinah	75.88	
	East of Madinah	73.59	
	West of Madinah	66.92	
	Central Region	73.41	

Table 5 shows the differences between mean scores and total scores on the QoL per the DLQI among demographic variables of the acne vulgaris sample. There was a significant difference between the mean score and QoL due to the different incomes of the patients (p = 0.013). Post hoc comparisons found there was a significant difference in the mean rank of the QoL scores between incomes, i.e., 6000-8000 SR on one side and less than 3000 SR and 3500-6000 SR on the other side (p = 0.002 and p = 0.003, respectively), favoring patients with incomes of 6000-8000 SR. There was a significant difference between incomes of 6000-8000 SR and more than 8000 SR (p = 0.039), favoring patients with incomes greater than 8000 SR.

**Table 5:** The differences between the mean scores per the DLQI among sociodemographic variables (n = 141) ^a^ Mann-Whitney U test, ^b^ Kruskal-Wallis test, p-value < 0.05 is statistically significant DLQI: Dermatology life quality index, SR: Saudi riyal

Variables	Category	Mean rank	p-value
Gender ^a^	Male	83.11	0.051
	Female	67.30	
Age ^b^	16 to 20	61.28	0.105
	21 to 25	75.55	
	26 to 30	77.95	
Social status^ a^	Single	68.81	0.265
	Married	77.64	
Educational level ^a^	Secondary education	70.88	0.980
	University	71.06	
Occupation status ^b^	Student	66.48	0.152
	Employee	83.57	
	Unemployed	77.17	
Income ^b^	Less than 3500 SR	69.01	0.013
	3500-6000 SR	56.97	
	6500-8000 SR	105.38	
	More than 8000 SR	71.88	
Area of residence^ b^	North of Madinah	83.12	0.076
	South of Madinah	67.09	
	East of Madinah	81.89	
	West of Madinah	73.37	
	Central Region	55.21	

Results of the vitiligo sample

The sample consisted of 30 Saudi patients with vitiligo residing in Madinah. Table 6 shows the demographic characteristics of the sample: 46.7% (n = 14) were male, 53.3% (n = 16) were female, and approximately 43% (n = 13) of the participants were between 16 and 20 years old. Most of the participants were single (80%, n = 24), students (56.7%, n = 17), and had an income of less than 3500 SR (70%, n = 21). Also, about 36.7% (n = 11) of the patients live in the west of Madinah, and the same percentage live in the east of Madinah as well. Besides, 96.7% (n = 29) of patients were non-smokers, and all patients had already visited a medical clinic.

**Table 6:** Sociodemographic characteristics of the vitiligo sample (n = 30) SR: Saudi riyal

Characteristics	Frequency	Percentages
Gender		
Male	14	46.7%
Female	16	53.3%
Age		
16 to 20	13	43.3%
21 to 25	9	30%
26 to 30	8	26.7%
Social status		
Single	24	80%
Married	6	20%
Educational level		
Secondary education	12	40%
University	18	60%
Occupation status		
Student	17	56.7%
Employee	7	23.3%
Unemployed	6	20%
Income		
Less than 3500 SR	21	70%
3500-6000 SR	2	6.7%
6500-8000 SR	5	16.7%
More than 8000 SR	2	6.7%
Area of residence		
North of Madinah	3	10%
South of Madinah	3	10%
East of Madinah	11	36.7%
Westof Madinah	11	36.7%
Central Region	2	6.7%
Clinic visit		
No	0	0%
Yes	30	100%
Smoking		
No	29	96.7%
Yes	1	3.3%

Table 7 describes the number and percentage of vitiligo patients according to their responses to the RSES. The mean of the total score for the sample on the scale was 13.7 (SD±4.1).

**Table 7:** Responses from and the mean for the vitiligo sample per the RSES (n = 30) RSES: Rosenberg’s self-esteem scale

Statements	Strongly agree		Agree		Disagree		Strongly disagree	
	N	%	N	%	N	%	N	%
1. On the whole, I am satisfied with myself.	1	3.3%	12	40%	15	50%	2	6.7%
2. At times I think I am no good at all.	5	16.7%	19	63.3%	6	20%	0	0%
3. I feel that I have a number of good qualities.	2	6.7%	20	66.7%	7	23.3%	1	3.3%
4. I am able to do things as well as most other people.	4	13.3%	16	53.3%	8	26.7%	2	6.7%
5. I feel I do not have much to be proud of.	5	16.7%	16	53.3%	8	26.7%	1	3.3%
6. I certainly feel useless at times.	5	16.7%	22	73.3%	0	0%	3	10%
7. I feel that I'm a person of worth, at least on an equal plane with others.	2	6.7%	17	56.7%	10	33.3%	1	3.3%
8. I wish I could have more respect for myself.	13	43.3%	17	56.7%	0	0%	0	0%
9. All in all, I am inclined to feel that I am a failure.	0	0%	14	46.7%	14	46.7%	2	6.7
10. I take a positive attitude toward myself.	2	6.7%	19	63.3%	8	26.7%	1	3.3%
The mean of the total score (±SD)	13.7 (± 4.1)							

**Figure 3:**
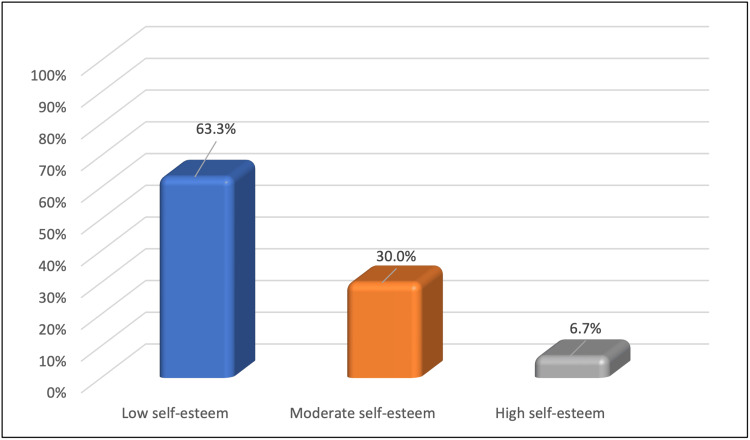
Levels of self-esteem in the vitiligo sample (n = 30)

Figure 3 demonstrates the distribution of the levels of self-esteem in the vitiligo sample. Around 63.3% (n = 19) of the participants had low self-esteem, while 30% (n = 9) of them had a medium rating, and 6.7% (n = 2) had high self-esteem.

Table 8 shows the responses of vitiligo patients to the DLQI. The mean of the total score for the sample on the scale was 15.2 (SD±5.8). 

**Table 8:** Responses from and the mean for the vitiligo sample per the DLQI (n = 30) DLQI: Dermatology life quality index

Questions	Very much		A lot		A little		Not at all OR Not relevant	
	N	%	N	%	N	%	N	%
Over the last week, how itchy, sore, painful or stinging has your skin been?	2	6.7%	5	16.7%	9	30.0%	14	46.7%
Over the last week, how embarrassed or self-conscious have you been because of your skin?	8	26.7%	16	53.3%	5	16.7%	1	3.3%
Over the last week, how much has your skin interfered with you going shopping or looking after your home or garden?	5	16.7%	15	50%	6	20%	4	13.3%
Over the last week, how much has your skin influenced the clothes you wear?	13	43.3%	13	43.3%	1	3.3%	3	10%
Over the last week, how much has your skin affected any social or leisure activities?	10	33.3%	14	46.7%	4	13.3%	2	6.7%
Over the last week, how much has your skin made it difficult for you to do any sport?	8	26.7%	11	36.7%	5	16.7%	6	20%
Over the last week, how much has your skin created problems with your partner or any of your close friends or relatives?	1	3.3%	14	46.7%	10	33.3%	5	16.7%
Over the last week, how much has your skin caused any sexual difficulties?	0	0%	0	0%	0	0%	30	100%
Over the last week, how much of a problem has the treatment for your skin been, for example by making your home messy, or by taking up time?	1	3.3%	24	80%	1	3.3%	4	13.3%
Over the last week, has your skin prevented you from working or studying?	No/Not relevant				Yes			
	26		86.7%		4		13.3%	
If "yes", over the last week how much has your skin been a problem at work or studying?			5	19.2%	14	53.8%	7	26.9%
The mean of the total score (±SD)	15.2 (±5.8)							

**Figure 4:**
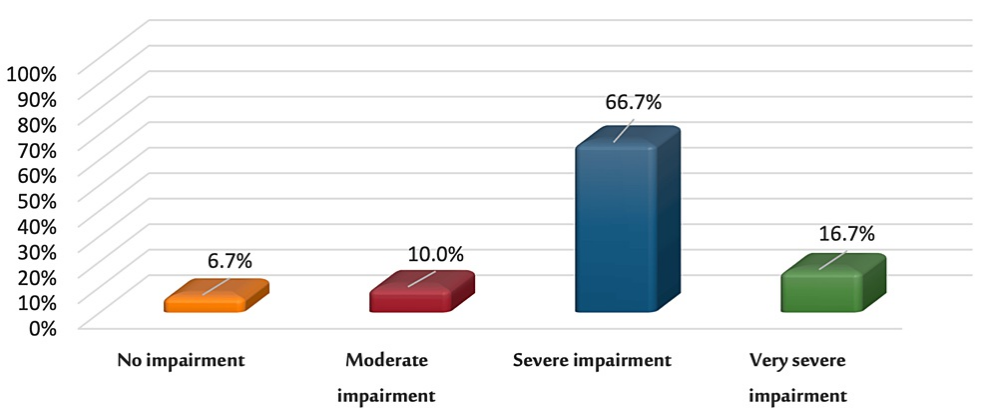
Levels of QoL in the vitiligo sample (n = 30) QoL: Quality of life

Figure 4 shows the distribution of the vitiligo sample to QoL levels per the DLQI. Of the vitiligo patients, 6.7% (n = 2) reported that vitiligo did not affect their lives, 10% (n = 3) said that it had a moderate effect, 66.7% (n = 20) stated that it had a severe effect, and 16.7% (n = 5) reported that vitiligo had a very severe effect on their lives.

Table 9 shows the differences between the mean scores of the RSES among demographic variables of the vitiligo sample. There was a significant difference between the mean score in the secondary stage and university according to the variable educational level (p <0.001). Persons with university education showed high self-esteem (15.61) compared to persons with secondary education (10.36).

**Table 9:** The differences between mean scores of RSES among demographic variables (n = 30) ^a ^Mann-Whitney U test, ^b ^Kruskal-Wallis test, ^c^ T-test, ^d^ one way ANOVA p-value < 0.05 is statistically significant RSES: Rosenberg’s self-esteem scale, SR: Saudi royal

Variables	Category	Mean rank	p-value
Gender^ a^	Male	17.79	0.181
	Female	13.50	
Age ^d^	16 to 20	16.83	0.232
	21 to 25	12.44	
	26 to 30	15.88	
Social status ^c^	Single	13.58	0.765
	Married	14.17	
Educational level ^c^	Secondary education	10.36	0.001
	University	15.61	
Occupation status ^d^	Student	14.06	0.199
	Employee	14.71	
	Unemployed	19.00	
Income ^b^	Less than 3500 SR	14.14	0.446
	3500-6000 SR	19.00	
	6500-8000 SR	16.50	
	More than 8000 SR	23.75	
Area of residence ^b^	North of Madinah	16.50	0.528
	South of Madinah	15.33	
	East of Madinah	18.36	
	West of Madinah	11.95	
	Central Region	18.00	

Table 10 reveals that there was an insignificant difference between the mean score and QoL among the demographic variables of the vitiligo sample.

**Table 10:** The differences between mean scores on DLQI among sociodemographic variables (n = 30) ^a ^T-test, ^b ^one-way ANOVA. A p-value < 0.05 is statistically significant. DLQI: Dermatology life quality index, SR: Saudi riyal

Variables	Category	Mean rank	p-value
Gender ^a^	Male	16.07	0.452
	Female	14.44	
Age ^b^	16-20	16.69	0.459
	21-25	14.17	
	26-30	15.88	
Social status^ a^	Single	16.50	0.572
	Married	15.33	
Educational level ^a^	Secondary education	16.58	0.460
	University	15.12	
Occupation status ^b^	Student	15.87	0.190
	Employee	14.71	
	Unemployed	19.00	
Income ^b^	Less than 3500 SR	16.75	0.622
	3500-6000 SR	17.00	
	6500-8000 SR	15.60	
	More than 8000 SR	12.50	
Area of residence^ b^	North of Madinah	14.67	0.880
	South of Madinah	18.67	
	East of Madinah	15.00	
	West of Madinah	15.64	
	Central Region	16.50	

## Discussion

The impact of acne vulgaris

Acne vulgaris, a common skin condition, is a serious health risk for teenagers and young adults. According to the literature, acne vulgaris is common, with a prevalence ranging from 39.9% to 96%, depending on the age group and population studied [11]. Acne vulgaris has a negative impact on DLQI and is linked to psychiatric morbidity. Emotional stress can aggravate acne, and acne patients can develop psychiatric problems as a result of their condition [12]. This study included 141 Saudi patients with acne vulgaris. Females outnumbered males (76.6% (n = 108) versus 23.4% (n = 33), which was consistent with other studies [13-16], possibly due to hormonal fluctuations during menstruation or a higher level of stress in females.

The age range of patients with acne vulgaris varies in various studies. The majority of studies included participants aged 13 to 18 years [14,17,18], as well as other studies from the previous 11 years [15,19,20] and some of the research from the previous 17 years [21]. The participants in this study ranged from 16 to 30 years old. The peak age for acne vulgaris is 17 years old [19]. Late adolescents, those over the age of 16, transition into young adult roles, and appearance becomes more important than at an earlier age [13]. The majority of participants in this study (43%, n = 61) were between the ages of 21 and 25, which is consistent with other studies, as acne develops during adolescence and subsides during adulthood [16,22].

Furthermore, the majority of participants were single (75.2%, n = 106), students (66.7%, n = 94), and had a monthly income of 3500 SR (70.9%, n = 100). Furthermore, 5.7% (n = 8) of participants were smokers. Only 62% (n = 88) of patients in this study visited a medical clinic. In this regard, Vilar et al. reported that only 5% of the adolescents with acne vulgaris in their study sought medical care for specific treatment [23]. We emphasize the importance of this information because, despite the fact that this dermatological disease is common, it has severe forms of presentation, affects QoL, and has psychological effects, and the majority of those affected do not seek medical care.

The high prevalence of inappropriate behavior, such as repeated manipulation of lesions, use of inappropriate products, and self-medication, suggests a lack of medical knowledge about skin care. Aside from a lack of access, another factor is social, as many patients who had appointments did not follow their care according to medical guidelines due to financial constraints. These issues serve as a wake-up call to the need for education initiatives and expanded access to acne vulgaris treatment within the healthcare system [23].

The current study's findings show that acne vulgaris has a negative impact on patients' DLQI and self-esteem. Self-esteem is defined as a positive or negative attitude toward oneself [24]. In today's society, where social media places a high value on appearance and body image, assessing self-esteem is more important [25]. Teenagers and young adults face significant pressure to maintain acceptable societal appearance standards. Acne and the imperfections that accompany it have been linked to depressive symptoms, humiliation, a sense of helplessness, a negative attitude, and diminished pride, self-worth, and body satisfaction in their adolescent and late adolescent years [26].

Acne can cause serious psychological problems. Patients with acne are more likely to have low self-esteem, low self-confidence, and social dysfunction, which can lead to anxiety, sadness, and occasionally suicidal ideation [16]. The mean total score of RSES for acne patients in this study was 20.3, with 5% (n = 7) of participants having low self-esteem, 48.2% (n = 68) having medium self-esteem, and 46.8% (n = 66) having high self-esteem. Many studies have found that acne patients have low self-esteem [27-30]. Akinboro et al. found that 1.5% of people had low self-esteem, while Nguyen et al. reported 46.8% [31,32].

Individuals with low self-esteem have feelings of inadequacy, incompetence, and inability to deal with challenges, while those with average self-esteem have oscillations between feelings of self-approval and rejection, and those with high self-esteem have self-judgment of value, competence, and trust [24]. Acne vulgaris can lead to low self-esteem, self-consciousness, and an impaired social life, as well as problems with daily activities [33,34].

Teixeira et al. also highlights the difficulty in predicting the true impact of acne vulgaris on self-esteem because it is influenced by several factors such as age, basal self-esteem, family support, and subjacent psychiatric pathology [35]. Alanazi et al. discovered that 29.1% of acne vulgaris cases had no impact, 56.3% had a small to moderate impact, and 14.5% had a large impact [3]. In Riyadh, Al Robaee discovered that the majority of cases (82.6%) reported depression as a result of the appearance of acne, more than half (57.8%) reported an impact on social relations, and 54.2% reported an impact on self-confidence [36]. Other studies revealed that acne has serious effects on body image, self-esteem, and socialization and may lead to feelings of anxiety, anger, depression, and social dysfunction [37,38].

According to the findings of this study per the RSES for acne, females had significantly higher self-esteem than males [15,28,39]. Females outperformed males in studies conducted by Cotterill et al., Halvorsen et al., and Ismail et al. Meanwhile, Durai and Nair discovered no gender differences in the scores, indicating that both men and women were concerned about their acne [16]. Dalgard et al. discovered that acne was associated with poor self-attitude and self-worth in girls on its own, independent of BMI and depression [40]. Self-esteem was found to be proportional and in direct correlation with the severity of acne, with participants with severe acne being more likely to be neurotic and have lower self-esteem [32,41]. 

The DLQI score for acne vulgaris patients in this study was 5.4, which was lower than studies by Ghaderi et al. (8.18), Chowdary et al. (7.84), Durai and Nair (6.91), but higher than Jancovick et al. (4.35), and Takahashi et al. (3.99) [42,43,16,14,44]. Alanazi et al. reported a mean DLQI score of 5.26 in Saudi Arabia. This finding is consistent with the findings of an Iranian study, which found that acne affected 51.8% of people's QoL, with a mean DLQI score of 6.42 [3,45]. In contrast, in a study conducted by Abdel-Hafez et al. in Egypt, the mean DLQI score in female and male subjects was 11.9 and 15.0, respectively [46]. In a study by Walker, performed on students in Scotland, the mean scores of the DLQI and the Cardiff acne disability index (CADI) were 1.7 and 1.9, respectively [18].

In this study, 30.5% (n = 47) of patients reported that acne had no effect on their lives, while 29.1% (n = 45) reported that acne had a mild effect, 23.4% (n = 35) reported a moderate effect, 5.6% (n = 10) reported a severe effect, and only 1.4% (n = 4) reported that acne had a very severe effect. The disparity between the current study's findings and those of other studies could be due to socioeconomic or cultural differences.

This study found a significant difference between the mean DLQI score and QoL in acne patients with different income levels. Post hoc comparisons revealed a significant difference in the mean rank of DLQI scores with the highest income (6000 to 8000 SR). In this regard, Arruhaily et al. used the CADI to assess the effects of acne on QoL. They discovered that neither the location of the acne nor demographic information (age, mitral status, college, residency, and BMI) were related to the CADI score [47].

They also discovered a statistically significant positive relationship between the CADI score and monthly income. A Malaysian study discovered a strong link between monthly income and CADI, yielding similar results [48]. In general, there was no correlation between the DLQI score and gender in most previous studies [16,43,44,49], which is consistent with our finding that both genders were concerned about their appearance and self-reported acne. However, Abdel-Hafez et al. reported that the higher mean DLQI scores of male acne patients than female patients may be explained by the fact that, in Egypt, women may be less exposed to social embarrassment than men because they cover their faces and the majority of them stay at home (housewives) [46]. While others reported that females showed higher DLQI scores versus males, this may be because of the significant gender difference in body-image recognition among females and male adolescents. Also, they reported that DLQI scores were significantly higher in those aged between 18 and 21 when compared to those between 22 and 30 years [15,28,42]. The discrepancy between the current study's findings and those of other studies could be due to differences in the duration and severity of acne. Ismail et al. discovered a link between age and DLQI, with most patients aged 21 to 25 years having higher scores [15]. Several other studies [13,17,50,51] have found that the negative impact increases with age.

Some studies [16,19,20] found that acne had a significant impact on DLQI based on occupation type. In contrast, no association between occupation and the DLQI scores was found in a study conducted in Greece [15]. In some studies [16,50], the level of education had no effect on the DLQI. According to some studies, an individual's marital status influences DLQI due to their cosmetic appearance. The link between smoking and acne is unknown. Nicotine's effect on nicotinic cholinergic receptors may be responsible for its pathogenesis [52].

The presence of acetylcholine receptor (AChR) and nicotinic activity in the pilosebaceous unit's infundibulum can promote infundibular epithelial hyperplasia and follicular plugging, implying a role for the cholinergic system in acne vulgaris [53]. Another mechanism is that arachidonic acid and polycyclic aromatic hydrocarbons in cigarette smoke stimulate the phospholipase A2-dependent inflammatory pathway [54]. There was no link found between smoking and acne. Eleni et al. and Mills et al. [13,55] discovered a negative correlation between smoking and acne. As a result, they proposed that some cigarette component, possibly nicotine, has an anti-inflammatory effect on acne. A study by Schäfer et al. found a link between smoking and acne [56].

The differences between studies highlight the importance of racial influences, population characteristics, study settings, and questionnaire design. The varied pattern of acne's impact on the DLQI aids us in understanding how an individual may be affected. The severity of acne and the individual's perception both contribute to the worsening of DLQI. The difference could be attributed to the lack of acne-specific measures, the limited range of scales used, the lack of a consistent acne grading system, self-evaluation of acne severity, different sample sizes, wide age ranges, and the fact that some studies use instruments whose questions are independent of skin disease severity or contain questions that strictly refer to skin disease symptoms rather than QoL.

The impact of vitiligo

This study included 30 Saudi patients with vitiligo. Demographic characteristics of vitiligo patients showed that females were slightly higher than males (53.3% (n = 16) versus 46.7% (n = 14)), and approximately 43.0% (n = 13) of patients were aged between 16 and 20 years old. Most of the participants were single (80.0%, n = 24), and students (56.7%, n = 17) had an income of <3500 SR (70.0%, n = 21). Besides, 3.3% (n = 1) of patients were smokers, and all patients had already visited a medical clinic.

Other studies, too, have reported that female participants outnumbered males [57-59]. Women are more embarrassed and self-conscious about the disease, so they may seek treatment more frequently than men. The mean RSES total score for vitiligo patients in this study was 13.7, indicating low self-esteem. In terms of self-esteem, 63.3% (n = 19) of vitiligo patients had low self-esteem, 30% (n = 9) had medium self-esteem, and 6.7% (n = 2) had high self-esteem. Self-esteem is defined as an individual's perception of themselves as adequate, valuable, effective, diligent, and successful [60]. Reduced self-esteem and DLQI were observed among vitiligo patients in a prior study that evaluated DLQI and RSES in 644 patients [61]. Khattri et al. reported a reduced mean self-esteem score in vitiligo patients [62].

Another study with 145 vitiligo patients revealed that consistent psychiatric monitoring decreased depressive symptoms and enhanced self-esteem [63]. In this study, the self-esteem score rank was higher in university students than in those with secondary education, which reflects that education affects levels of self-esteem. In our study, the mean responses of vitiligo patients to DLQI were 15.2 ± 5.8 SD. We found that 6.7% (n = 2) reported that vitiligo did not affect their lives, 10% (n = 3) said it had a moderate effect, 66.7% (n = 20) stated it had a severe effect, and 16.7% (n = 5) reported that vitiligo had a very severe effect on their lives. The mean score obtained by this study was higher than those in the three previous studies (9 ± 6.5 SD, 14.72 ± 5.173 SD, and 5.64 ± 5.2 SD) conducted in Saudi Arabia, which indicates a severe effect on patients’ lives [64-66]. Globally, DLQI mean scores range from 1.82 to 15, with a total mean DLQI score of 8.2, which indicates a moderate effect [67]. However, our finding was higher than the mean DLQI score when compared with 4.82 [61], 4.95 [25], 7.2 [59], and 10.67 [68] reported in other studies.

The mean score for DLQI differed insignificantly across demographic variables in the vitiligo sample. The difference in QoL could be attributed to social acceptability and how society or family members react to the disease. Others reported that DLQI affected females more than males [69-73], which contradicts the findings of Zandi et al. and Mishra et al., who reported that there was no statistically significant difference between men and women in DLQI [74,75].

Females may have more impairment because they are more cosmetically conscious, and females bear a greater social burden because they still face more discrimination in society than males [68,76]. The stark contrast of vitiligo lesions in dark-skinned people may be to blame for the negative impact on QoL; similarly, cultural factors can do the same. Patients with vitiligo, like patients with leprosy, are considered unclean and unfit for marriage in many parts of India [77]. There were no associations found in this study between the DLQI score and patient demographic characteristics. Ongenae et al. reported a significant correlation between DLQI score, gender, and previous treatment in this regard [25]. However, no correlation with patient age was found, as in our study. In Iran, Dolatshashi et al. discovered that the visibility of lesions was not significantly related to the DLQI score [78]. Elgazzar discovered a statistically significant relationship between the DLQI score and marital status in Saudi Arabia [76]. Divorced patients had a greater effect on the DLQI score than single patients. Dolatshahi et al. also found a link between marital status and DLQI [78]. Furthermore, Elgazzar and Bae et al. found that advanced education was significantly related to DLQI [76,79].

Silpa-Archa et al. found a trend of higher mean DLQI scores among those who worked versus those who were students, unemployed, or retired, but there was no statistical significance between groups [80]. Wong and Baba's study, on the other hand, found that patients who worked had significantly higher DLQI scores than those who were retired. People who were employed or had to go out for work and meet new people may have felt more embarrassed, resulting in a decrease in DLQI [81]. The difference in QoL may be related to social acceptability and how society or family members react to the disease.

Limitations

The study population was limited to patients in Madinah and was drawn at random, so it cannot be generalized to other parts of Saudi Arabia. Only the RSES and the DLQI questionnaire were used to assess self-esteem and quality of life, and they were able to detect psychosocial problems but not depression or anxiety without clinical assessment. Furthermore, because the questionnaire is self-reported, disease severity was not assessed, and the small sample size highlights the study's limitations.

## Conclusions

Our research confirms that acne vulgaris and vitiligo have a negative impact on self-esteem and quality of life. Gender and monthly income have an impact on self-esteem and DQLI in acne vulgaris patients, whereas education levels have an impact on self-esteem in vitiligo patients. Along with medical treatment, effective treatment and the psychological improvement of the patient should be prioritized. When QoL and self-esteem are impaired, an appropriate referral to a psychologist is required.
